# [4-(2-Aminoethyl)morpholine-κ^2^ *N*,*N*′]di­bromidocadmium(II): synthesis, crystal structure and Hirshfeld surface analysis

**DOI:** 10.1107/S2056989024000963

**Published:** 2024-02-08

**Authors:** B. Chidambaranathan, S. Sivaraj, P. Vijayamathubalan, S. Abraham Rajasekar, S. Selvakumar

**Affiliations:** a PG and Research Department of Physics, Government Arts College for Men (Autonomous), Nandanam, Chennai 600 035, Tamil Nadu, India; b Department of Physics, Sir Theagaraya College, Old Washermanpet, Chennai 600 021, Tamil Nadu, India; Universidad Nacional Autónoma de México, México

**Keywords:** crystal structure, morpholine ligand, Hirshfeld surface analysis, FTIR, NMR

## Abstract

The title compound, [CdBr_2_(C_6_H_14_N_2_O)], was synthesized upon complexation of 4-(2-aminoethyl)morpholine and cadmium(II) bromide tetra­hydrate at 303 K. It crystallizes as a centrosymmetric dimer, with one cadmium atom, two bromine atoms and one *N*,*N′*-bidentate 4-(2-aminoethyl)morpholine ligand in the asymmetric unit. The metal atom is six-coordinated and has a distorted octa­hedral geometry. In the crystal, O⋯Cd inter­actions link the dimers into a polymeric double chain and inter­molecular C—H⋯O hydrogen bonds form *R*_2_^2^(6) ring motifs. Further C—H⋯Br and N—H⋯Br hydrogen bonds link the components into a three-dimensional network. As the N—H⋯Br hydrogen bonds are shorter than the C—H⋯Br inter­actions, they have a larger effect on the packing. A Hirshfeld surface analysis reveals that the largest contributions to the packing are from H⋯H (46.1%) and Br⋯H/H⋯Br (38.9%) inter­actions with smaller contributions from the O⋯H/H⋯O (4.7%), Br⋯Cd/Cd⋯Br (4.4%), O⋯Cd/Cd⋯O (3.5%), Br⋯Br (1.1%), Cd⋯H/H⋯Cd (0.9%), Br⋯O/O⋯Br (0.3%) and O⋯N/N⋯O (0.1%) contacts.

## Abstract 2

The title coordination compound was synthesized upon complexation of 4-(2-aminoethyl)morpholine and cadmium(II) bromide tetra­hydrate at 303 K. It crystallizes as a centrosymmetric dimer, with one cadmium atom, two bromine atoms and one *N*,*N′*-bidentate 4-(2-aminoethyl)morpholine ligand in the asymmetric unit.

## 1. Chemical context

Inorganic–metal halides may be associated with functionalized organic mol­ecules (for example carb­oxy­lic acids, amides or amines) to produce neutral or ionic coordination compounds that combine and change the properties of both components. Fine-tuning the stoichiometry, reaction conditions and geometry of the organic ligands allows control of the dimensionality and geometry of the final product, resulting in a wide range of systems (Constable, 2019 ▸). This has become the main focus of coordination chemistry and has allowed for the development of many research fields, such as medicinal chemistry of coordination compounds, homogenous catalysis, and metal-organic frameworks (Malinowski *et al.* 2020 ▸; Zecchina & Califano 2018 ▸; Yaghi *et al.* 2019 ▸; Jones & Thornback 2007 ▸). In this context, morpholine is a heterocyclic bidentate ligand frequently used in medicinal chemistry and a privileged structural component of bioactive mol­ecules. The morpholine mol­ecule has become one of the most promising moieties evaluated in structure-activity relationship (SAR) studies, as it induces biological activity, as well as an improved pharmacokinetic and metabolic profile to the biomolecules that contain it. Morpholine and its derivatives have long been known for various activities such as analgesic, anti-inflammatory, anti­oxidant, anti­cancer, anti-neurodegenerative, *etc*. As a result of its biological and pharmacological importance, the synthesis of morpholine compounds has been extensively studied by many researchers (Rekka & Kourounakis 2010 ▸; Wijtmans *et al.*, 2004 ▸; Ilaš *et al.*, 2005 ▸; Pal’chikov 2013 ▸). Herein, we report the synthesis of the coordination compound [4-(2-aminoethyl)morpholine-κ^2^-*N*,*N*′]di­bromidocadmium(II) and examined it using single crystal X-ray diffraction, FTIR, NMR, and Hirshfeld surface studies as a part of our ongoing inter­est in morpholine derivatives. 
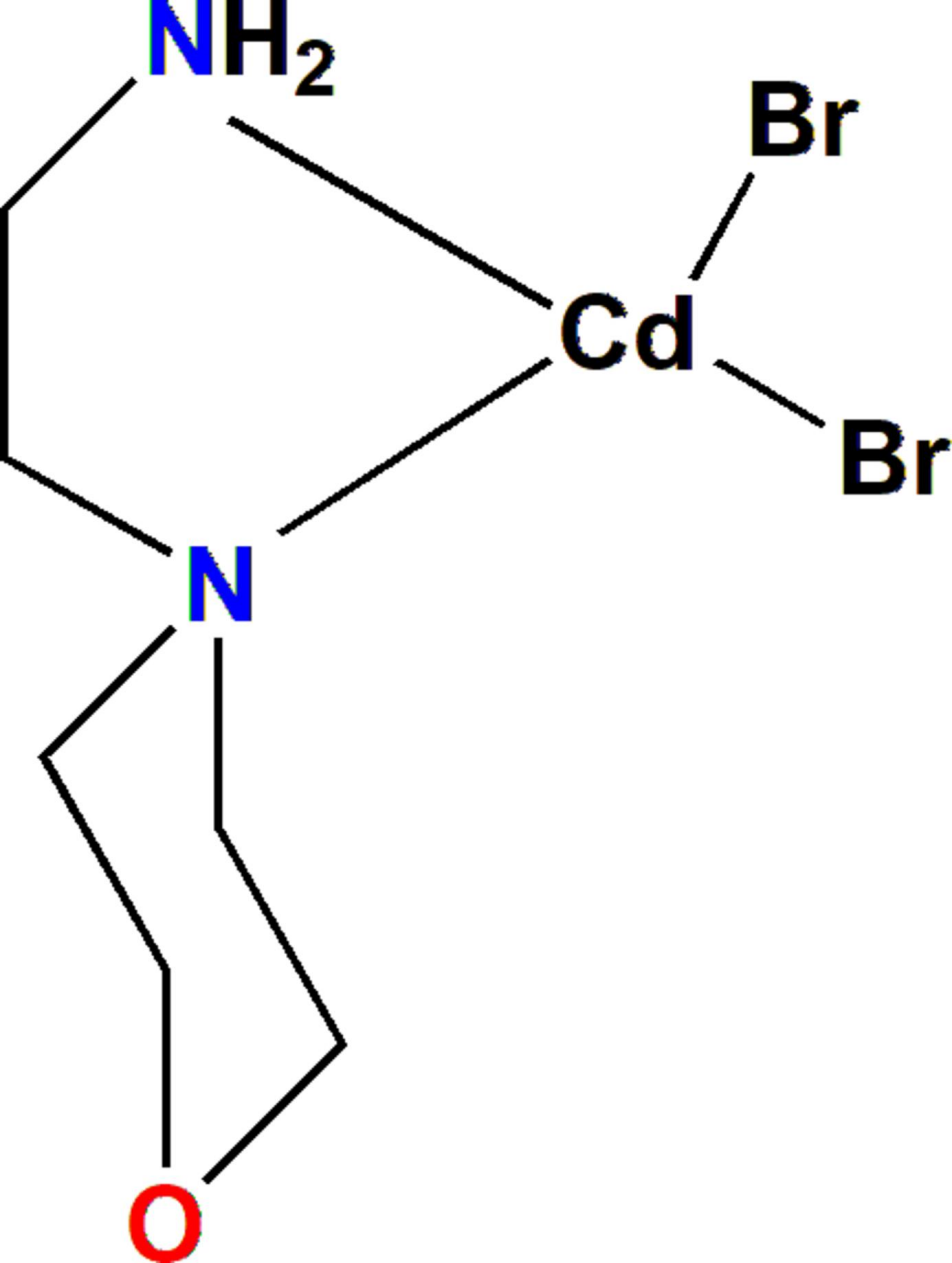


## 2. Structural commentary

**Figure 1:**
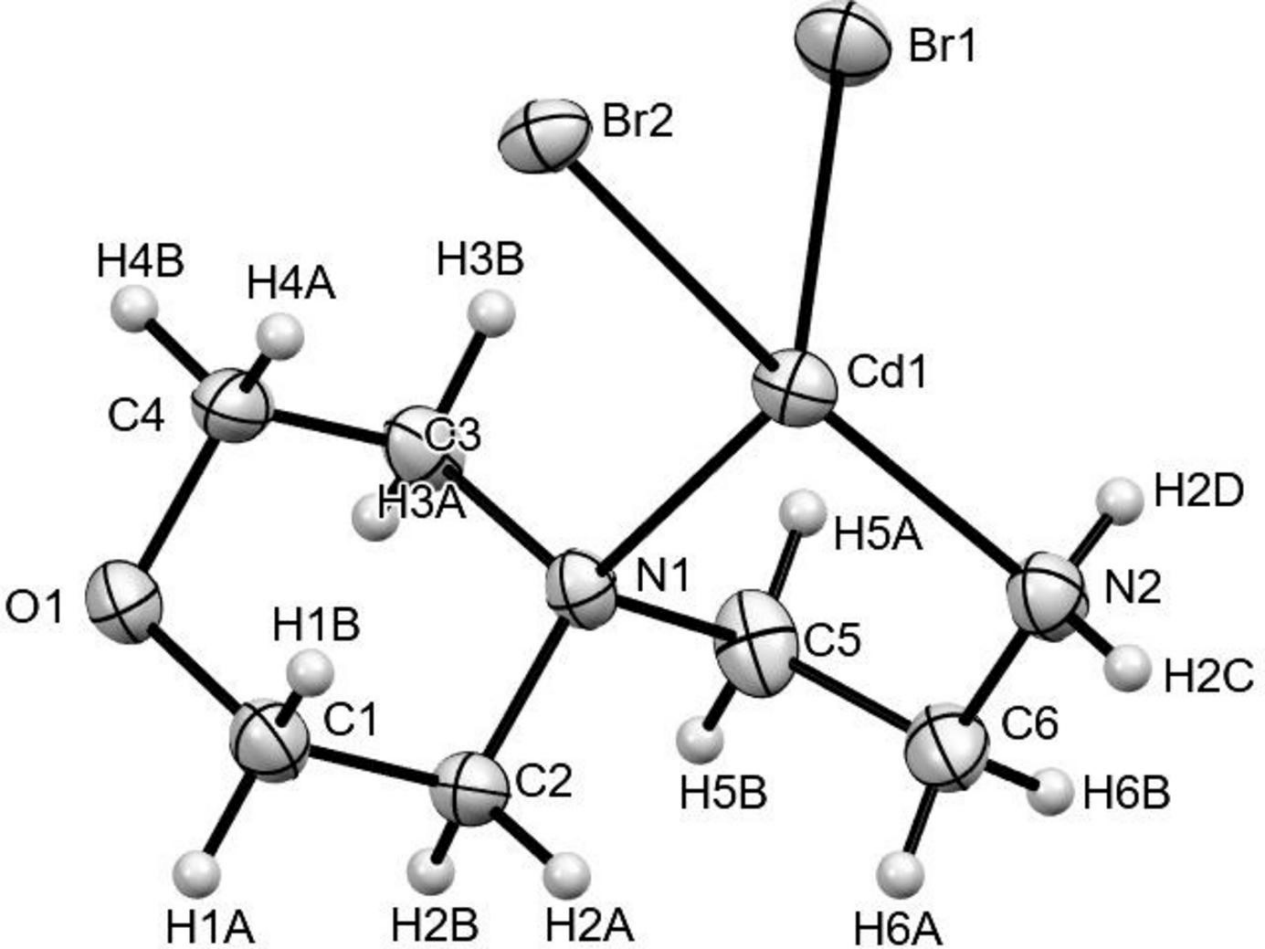
Ellipsoid plot of the title compound with displacement ellipsoids drawn at the 50% probability level.

The title compound crystallizes in the triclinic *P* 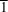 space group. Fig. 1 ▸ depicts a perspective view of the mononuclear centrosymmetric complex, [(Cd)(*L*)(Br)_2_], where *L* = 4-(2-aminoethyl)morpholine, with the atom-labeling scheme. The asymmetric unit contains half of the mol­ecule, consisting of one cadmium cation, two bromine anions and one 4-(2-aminoethyl)morpholine ligand that are located on a general positions and the other half of the mol­ecule is generated by inversion symmetry. Although the synthesis was carried out in water, the title compound is neither a hydrate nor is water present in the coordination sphere of the metal. If water enters the coordination sphere of cadmium, the resulting complex is usually ionic, as one Br^−^ has to stay outside the coordination sphere leading to lower entropy for the system. In addition, the large Br^−^ ion is a better bridging ligand than water and can link the components in a three-dimensional network. Hence, ignoring water during crystallization is more advantageous than retaining it in the coordination sphere.

**Figure 2:**
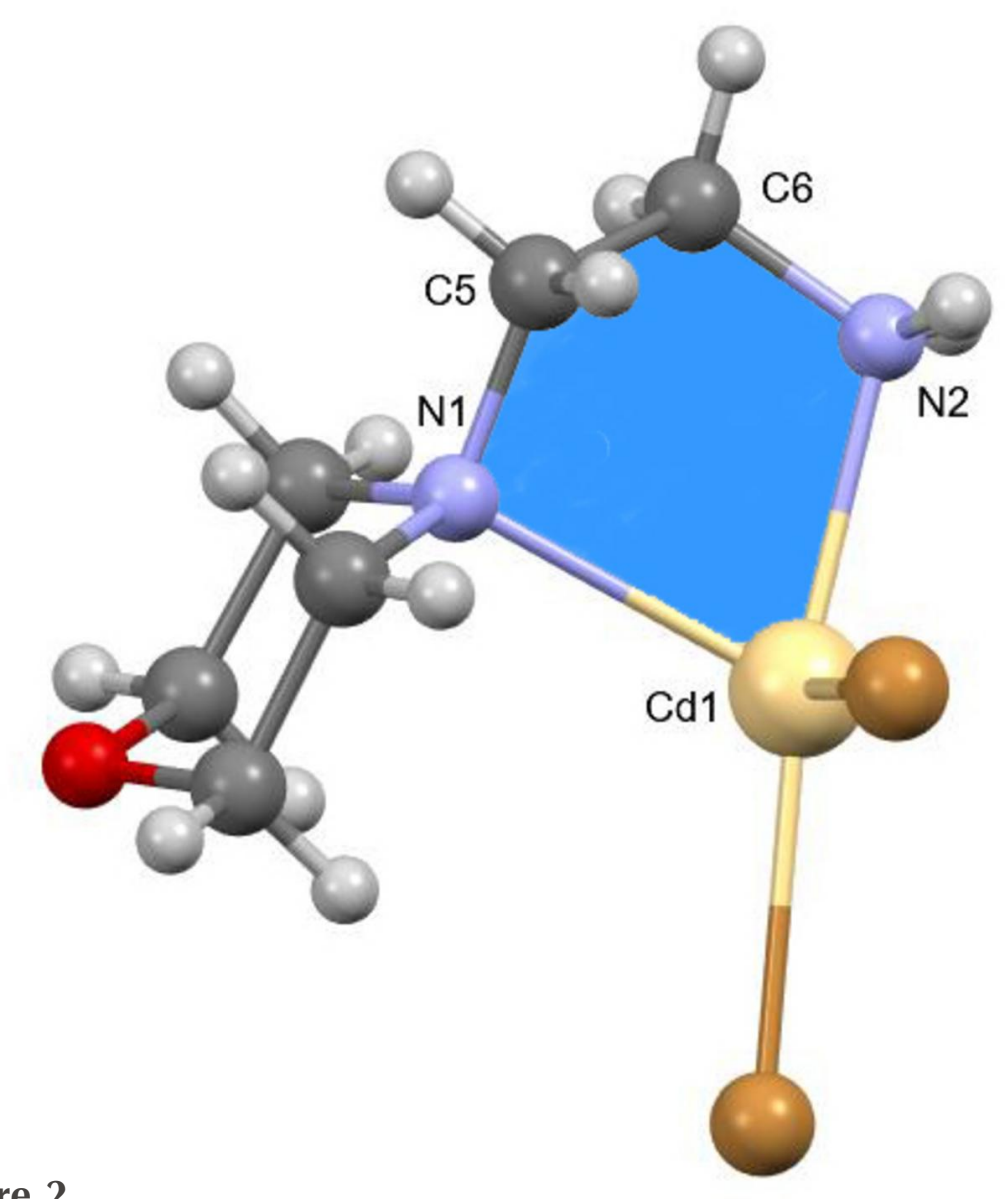
The five-membered chelate ring present in the title compound.

In the structure, one of the symmetry-independent bromine atoms (Br1) is terminal, while the other (Br2) bridges two cadmium atoms related by inversion (−*x* + 1, −*y*, −*z* + 1). The metal atom further coordinates the 4-(2-aminoethyl)morpholine in a *N*,*N′* bidentate fashion, forming a five-membered chelate ring (Cd1–N1–C5–C6–N2), which is shown in Fig. 2 ▸. The last coordination site of the distorted octa­hedron around the cadmium atom is occupied by an oxygen atom from a different morpholine moiety (*x*, *y* − 1, *z*). The size of the chelate ring is a key component in metal ion selection, with five-membered chelate rings preferring metal ions with an ionic radius near 1.0 Å. Baza­rgan *et al.* (2019 ▸) reported that the optimal size for the N—*M* distance is 2.5 Å and the N—*M*—N angle is 69° for five-membered N–C–C–N–*M* chelate rings. In five-membered chelate rings, the *M*—N bond lengths and the N—*M*—N bond angle are considered to be inversely linked (Hancock 1992 ▸; Hancock *et al.*, 2007 ▸; Dean *et al.*, 2008 ▸). The Cd1—N1 and Cd1—N2 distances are 2.504 (2) and 2.306 (3) Å, respectively, while the N1—Cd—N2 angle is 76.06 (8)°. This chelate ring pattern appears to be present in all reported structures of with a metal coordinated by 4-(2-aminoethyl)morpholine (Ikmal Hisham *et al.*, 2010 ▸; Suleiman Gwaram *et al.*, 2011 ▸). According to the structural data for the title compound, the torsion angles O1—C1—C2—N1 and N1—C3—C4—O1 of the morpholine ring are 55.6 (3) and −61.5 (3)°, respectively. These values are comparable with those reported for similar compounds such as *cis*-[4-(2-aminoethyl)morpholine-κ^2^ *N*,*N*′]di­chlorido­plati­num(II) (O1—C5—C6—N2 = 55° and N1—C3—C4—O1 = −59.9°; Shi *et al.* 2006 ▸) and bis­(acetato)­bis­[4-(2-aminoethyl)morpholine-κ^2^ *N*,*N*′]cadmium(II) tetra­hydrate (O3—C1—C2—N1 = 56° and N1—C4—C3—O3 = −59.6°; Chidambaranathan *et al.*, 2023*c* ▸). This validates the chair formation of morpholine rings, also observed in previously reported morpholine compounds (Konar *et al.*, 2005 ▸; Chattopadhyay *et al.*, 2005 ▸; Brayshaw *et al.*, 2012 ▸; Koćwin-Giełzak & Marciniak, 2006 ▸; Chidambaranathan *et al.*, 2023*a* ▸).

## 3. Supra­molecular features

**Figure 3:**
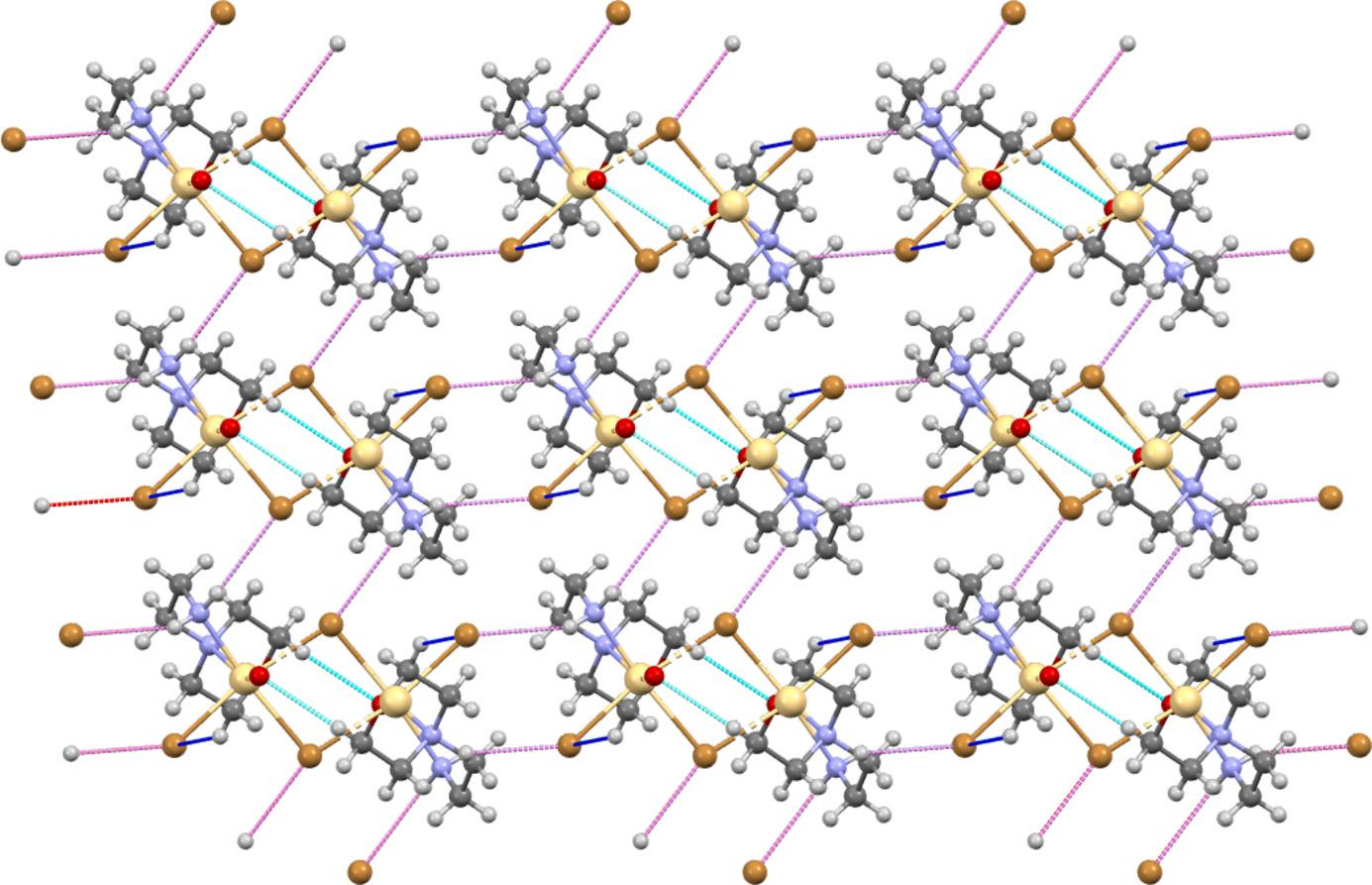
Packing diagram of the title compound along the *b*-axis.

**Figure 4:**
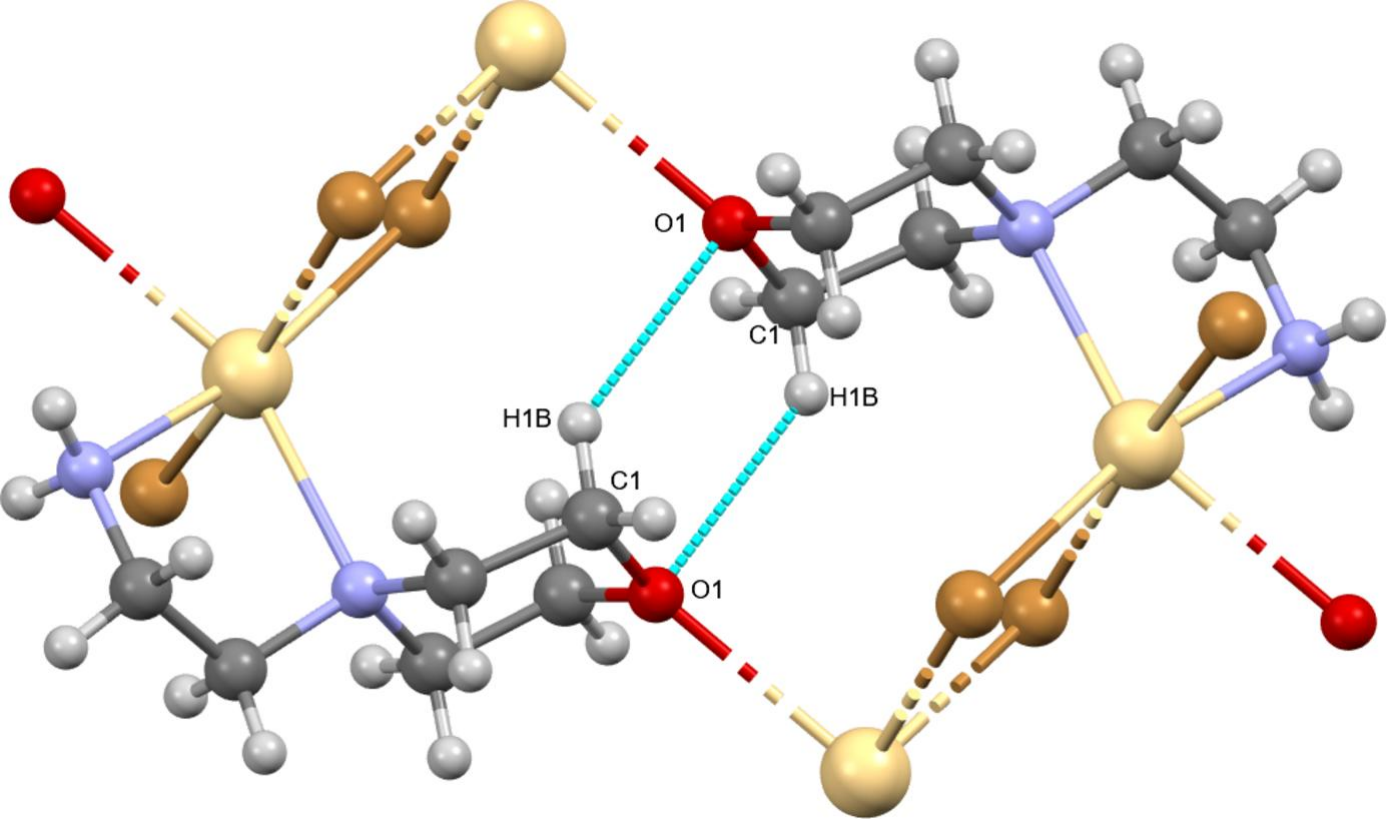
The 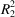 (6) motif formed by the inter­molecular inter­actions.

The morpholine mol­ecule is potentially an ambidentate N- and O-donor ligand, where the binding of morpholine to the metal center is most commonly accomplished through the nitro­gen atom (Cvrtila *et al.*, 2012 ▸; Cindric *et al.*, 2013 ▸), except in cases where the nitro­gen atom is protonated (Li *et al.*, 2010 ▸; Willett *et al.*, 2005 ▸). This leaves the oxygen atom free to participate in supra­molecular inter­connections *via* the formation of additional coordination bonds, acting as an acceptor for a halogen bond (Lapadula *et al.*, 2010 ▸) or participating in hydrogen bonding (Weinberger *et al.*, 1998 ▸), which can result in many different supra­molecular architectures. A packing diagram of the title compound along the *b*-axis is shown in Fig. 3 ▸, showing the inter­molecular C—H⋯O, C—H⋯Br and N—H⋯Br inter­actions (Table 1 ▸). The Br1 anion links adjacent mol­ecules along the *b*-axis direction *via* the H3*B* and H4*B* atoms of the morpholine ring. Similarly, the Br2 anion links adjacent mol­ecules along the *a*-axis direction *via* the H2*C* atom. The corresponding inter­action distances for H3*B*⋯Br1, H4*B*⋯Br1 (*x*, *y* + 1, *z*) and H2*C*⋯Br1 (*x* − 1, *y*, *z*) are 2.96, 2.91 and 2.95 (2) Å, respectively. Further C—H⋯Br and N—H⋯Br hydrogen bonds link the components into a three-dimensional network. Owing to the higher electronegativity of the N—H⋯Br hydrogen bonds, they are shorter than the C—H⋯Br ones and hence they will have a larger effect on the packing than the C—H⋯Br inter­actions. On the other hand, the O—Cd coordination bond contributes to the formation of the three-dimensional network more than the N—H⋯Br and C—H⋯Br hydrogen bonds. Fig. 4 ▸ shows the 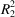 (6) ring motif formed between two mol­ecules through C—H⋯O inter­molecular inter­actions (Bernstein *et al.*, 1995 ▸; Motherwell *et al.*, 2000 ▸).

**Figure 5:**
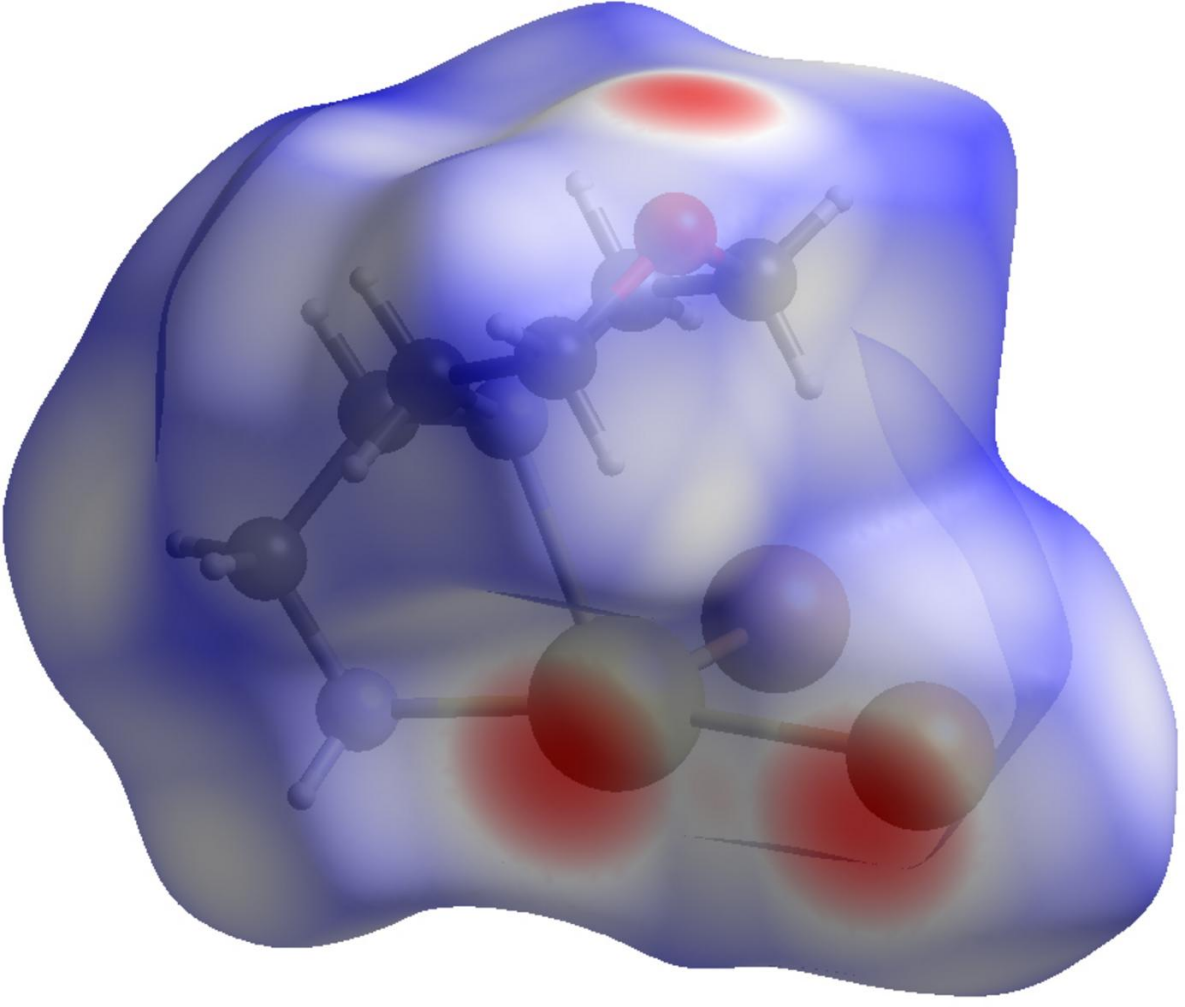
View of the Hirshfeld surface of the title compound mapped over *d*_norm_.

To examine the inter­molecular inter­actions present in the title compound in more detail, a Hirshfeld surface analysis was performed and the two-dimensional fingerprint plots were generated with *CrystalExplorer 21.5* (Spackman *et al.*, 2021 ▸). The three-dimensional *d*_norm_ surface is shown in Fig. 5 ▸. Here the white regions relate to contacts with distances equal to the sum of the van der Waals radii, red-colored regions indicate contacts with distances shorter than the sum of the van der Waals radii, while blue areas indicate distances longer than the sum of the van der Waals radii (Venkatesan *et al.*, 2016 ▸). This colored mapping of contacts allows the visual identification of regions susceptible to participating in inter­actions with other mol­ecules. Fig. 5 ▸ shows the most prominent inter­molecular inter­actions as red spots corresponding to the Cd—Br and Cd⋯O contacts.

**Figure 6:**
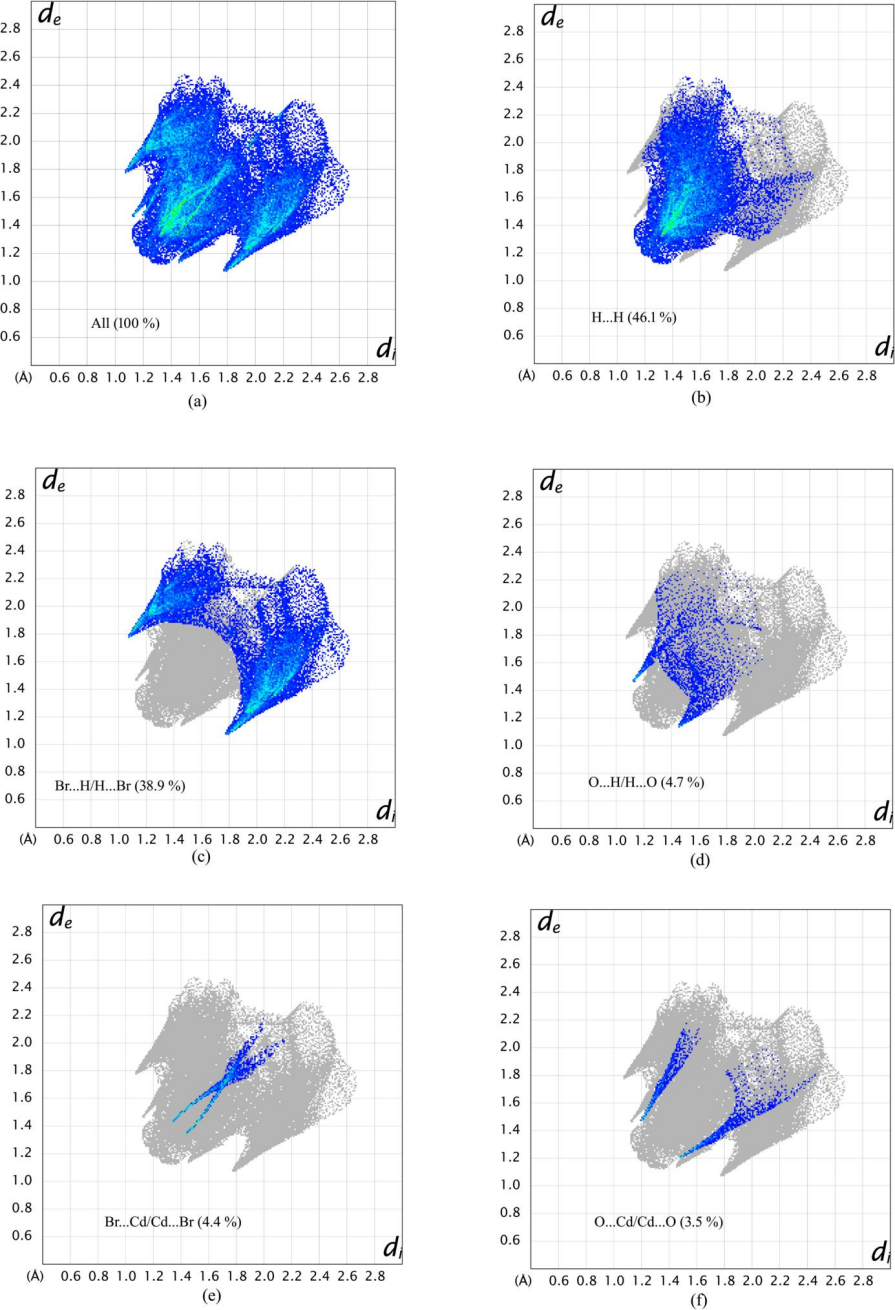
The two-dimensional fingerprint plots for the title compound showing (*a*) all inter­actions, and delineated into (*b*) H⋯H, (*c*) H⋯Br/Br⋯H, (*d*) H⋯O/O⋯H, (*e*) Cd⋯Br/Br⋯Cd and (*f*) O⋯Cd/Cd⋯O inter­actions.

The two-dimensional fingerprint plots are shown in Fig. 6 ▸. Each point of the Hirshfeld surface is associated with two types of distances: *d*_e_ is the distance from the point to the nearest-to-the-surface external nucleus and *d*_i_ is the distance from the point to the nearest-to-the-surface inter­nal nucleus. The normalized contact distance, *d*_norm_, is the sum of the van der Waals radii, *d*_e_ + *d*_i_, of each atom (McKinnon *et al.*, 2007 ▸; Hathwar *et al.*, 2015 ▸). The largest contributions to the Hirshfeld surface are represented as a point at *d*_e_ + *d*_i_ ∼2.4 Å due to H⋯H (46.1%), a pair of wings with the tip at *d*_e_ + *d*_i_ ∼2.85 Å due to H⋯Br/Br⋯H (38.9%), a pair of spikes at *d*_e_ + *d*_i_ ∼2.45 Å due to H⋯O/O⋯H (4.7%), a tip of a scissor-like image at *d*_e_ + *d*_i_ ∼2.7 Å due to Cd⋯Br/Br⋯Cd (4.4%) and a feather-like image at *d*_e_ + *d*_i_ ∼2.7 Å due to O⋯Cd/Cd⋯O (3.5%) contacts. The other contributions are Br⋯Br (1.1%), Br⋯O/O⋯Br (0.3%) and O⋯N/N⋯O (0.1%). All these inter­actions play a crucial role in the overall stabilization of the crystal packing.

## 4. Database survey

A search in the Cambridge Structural Database (CSD, version 5.40; Groom *et al.*, 2016 ▸) for the keyword ‘4-(2-amino­eth­yl)morpholine’ yielded 21 hits for coordination compounds with metals, including *trans*-bis­(iso­thio­cyanato-*N*)bis­[4-(2-amino­eth­yl)morpholine-κ^2^-*N*,*N′*]nickel(II) (NENSUU; Laskar *et al.*, 2001 ▸), (μ_2_-oxalato)-bis­[4-(2-amino­eth­yl)morpholine-κ^2^-*N*,*N′*]dicopper(II) (YIKQAK; Mukherjee *et al.*, 2001 ▸), *catena*-[bis­(μ_2_-dicyanamide-*N*,*N*′)-[4-(2-amino­eth­yl)morpholine-κ^2^-*N*,*N′*]nickel (II) (FIJROG; Konar *et al.*, 2005 ▸), bis­[4-(2-amino­eth­yl)morpholine-κ^2^-*N*,*N′*]copper(II) bis­(tetra­fluoro­borate) (RAPHEW; Sander *et al.*, 2005 ▸), [4-(2-amino­eth­yl)morpholine-κ^2^-*N*,*N′*]aqua­(oxalate-*O*,*O*′)-copper(II) monohydrate (XAZRUM; Koćwin-Giełzak & Marciniak, 2006 ▸), *trans*-bis­[4-(2-amino­eth­yl)morpholine-κ^2^-*N*,*N′*]-bis­(nitrito)nickel(II) (NAVNAA; Chattopadhyay *et al.*, 2005 ▸; RANVEJ and NAVNAA01; Brayshaw *et al.*, 2012 ▸), *cis*-di­chloro­[4-(2-amino­eth­yl)morpholine-κ^2^-*N*,*N′*]platinum(II) (WENQUC; Shi *et al.*, 2006 ▸), *cis*-(cyclo­butane-1,1-di­carboxyl­ato)-[4-(2-amino­eth­yl)morpholine-κ^2^-*N*,*N′*]platinum(II) trihydrate (TEVSAP and TEVSAP01; Xie *et al.*, 2007 ▸), bis­(5,5-di­ethyl­barbiturato-*N*)-[4-(2-amino­eth­yl)morpholine-κ^2^-*N*,*N′*]cop­per(II) (TUJRIA; Suat Aksoy *et al.*, 2009 ▸), *catena*-[(μ_4_-azido-*N* ^1^,*N* ^1^,*N* ^1^,*N* ^3^)-(μ_3_-azido-*N* ^1^,*N* ^1^,*N* ^1^)-tris­(μ_2_-azido-*N* ^1^,*N* ^1^,*N* ^1^)(μ_2_-azido-*N* ^1^,*N* ^3^)-[4-(2-amino­eth­yl)morpholine-κ^2^-*N*,*N′*]-tri-copper(II)] (IMETAW; Mukherjee & Mukherjee, 2010 ▸), tetra­carbonyl-[4-(2-amino­eth­yl)morpholine-κ^2^-*N*,*N′*]molybdenum(0) diglyme solvate (CIYBIX; Kromer *et al.*, 2014 ▸), bis­[4-(2-amino­eth­yl)morpholine-κ^2^-*N*,*N′*][5,10,15,20-tetra­kis(4-meth­oxy­phen­yl) porphyrinato]iron(II) (NABXEW; Ben Haj Hassen *et al.*, 2016 ▸; NABXEW01; Khelifa *et al.*, 2016 ▸), (1,1,1,4,4,4-hexa­fluoro-2,3-bis­(tri­fluoro­meth­yl)butane-2,3-dio­lato)-[4-(2-amino­eth­yl)morpholine-κ^2^-*N*,*N′*]-nitro­sylcobalt (DAPKOY; Popp *et al.*, 2021 ▸), di­chloro­bis­[4-(2-amino­eth­yl)morpholine-κ^2^-*N*,*N′*]cadmium(II) (ULAJEX; Suleiman Gwaram *et al.*, 2011 ▸), bis­[4-(2-amino­eth­yl)morpholine-κ^2^-*N*,*N′*]di­aqua­nickel(II) dichloride (VEPHIL; Chidambaranathan *et al.*, 2023*b* ▸) and bis­(acetate)-bis­[4-(2-amino­eth­yl)morpholine-κ^2^-*N*,*N′*]cadmium(II) tetra­hydrate (QEWKUC and FITXAL; Chidambaranathan *et al.*, 2023*c* ▸). All of these structures are consolidated by hydrogen bonding. As with the other metal complexes of 4-(2-amino­eth­yl)morpholine, the morpholine ring adopts a chair conformation, and the amine performs as an *N*,*N′*-bidentate ligand to form a five-membered chelate ring with the metal center.

## 5. Synthesis and crystallization

**Figure 7:**
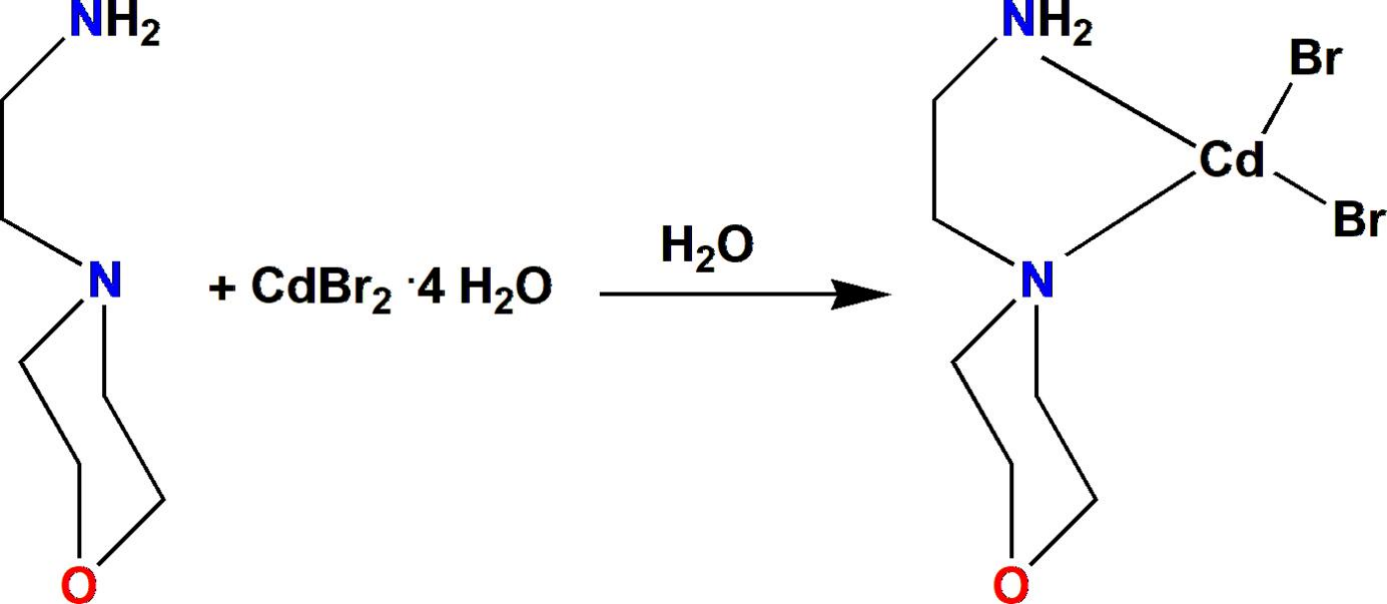
Synthesis of the title compound.

The reaction scheme is shown in Fig. 7 ▸. Cadmium bromide tetra­hydrate (3.44 g, 0.01 mol) and 4-(2-aminoethyl)morpholine (1.30 g, 0.01 mol) in a stoichiometric ratio of 1:1 were dissolved in double-distilled water at 303 K. The solvent was evaporated slowly at room temperature and plate-like orange single crystals were obtained after one week, m.p.: 497.5 K; yield: 78%; Elemental analysis for C_6_H_14_Br_2_CdN_2_O (402.41g·mol^−1^) theor(%): C, 17.91; H, 3.51; N, 6.96.; found(%): C, 16.98; H, 3.48; N, 6.42.

The FTIR spectrum of the title compound was recorded on a Bruker FTIR spectrometer. FTIR for title compound (KBr, cm^−1^): 3304 (*m*, N—H), 2950 (*w*, C—H), 1598 (*w*, C—N), 1454 (*s*, C—C), 1108 (*s*, C—N), 612 (*s*, *M*—N); FT–IR for free ligand (Edwin *et al.*, 2017 ▸); (KBr, cm^−1^): 3365 (*s*, N—H), 2954 (*s*, C—H), 1581 (*m*, C—N), 1456 (*s*, C—C), 1115 (*s*, C—N); ^1^H NMR (500 MHz. D_2_O, δ, ppm), 3.74 (*t*, 4H, –CH_2_—O—CH_2_), 2.92 (*t*, 4H, –CH_2_—N—CH_2_), 2.58 (broad singlet, 2H, N—CH_2_), 2.55 (*t*, 2H, –CH_2_—NH_2_).

## 6. Refinement details

Crystal data, data collections and structure refinement details are summarized in Table 2 ▸. All C–H atoms were positioned geometrically, C—H = 0.97 Å and refined as riding with *U*_iso_(H) = 1.2*U*_eq_(C). The acidic nitro­gen-bound protons H2*C* and H2*D* were localized from electron-density maps and refined freely with distance restraints (DFIX) and with *U*_iso_(H) = 1.2*U*_eq_(N).

## Supplementary Material

Crystal structure: contains datablock(s) I. DOI: 10.1107/S2056989024000963/jq2033sup1.cif

Structure factors: contains datablock(s) I. DOI: 10.1107/S2056989024000963/jq2033Isup4.hkl

CCDC reference: 2298040

Additional supporting information:  crystallographic information; 3D view; checkCIF report

## Figures and Tables

**Table 1:** Hydrogen-bond geometry (Å, °)

*D*—H⋯*A*	*D*—H	H⋯*A*	*D*⋯*A*	*D*—H⋯*A*
C1—H1*B*⋯O1^i^	0.97	2.59	3.370 (4)	138
C3—H3*B*⋯Br1	0.97	2.96	3.720 (3)	137
C4—H4*B*⋯Br1^ii^	0.97	2.91	3.678 (3)	137
N2—H2*C*⋯Br2^iii^	0.89 (2)	2.95 (2)	3.761 (3)	153 (3)
N2—H2*D*⋯Br1^iv^	0.87 (2)	2.86 (2)	3.628 (3)	149 (3)

Symmetry codes: (i) 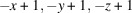 ; (ii) 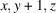 ; (iii) 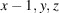 ; (iv) 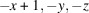 .

**Table 2:** Experimental details

Crystal data	
Chemical formula	[CdBr_2_(C_6_H_14_N_2_O)]
*M*_r_	402.41
Crystal system, space group	Triclinic, *P* 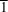
Temperature (K)	299
*a*, *b*, *c* (Å)	7.1291 (2), 7.1662 (2), 11.0151 (3)
α, β, γ (°)	77.704 (1), 80.079 (1), 72.371 (1)
*V* (Å^3^)	520.49 (3)
*Z*	2
Radiation type	Mo *K*α
μ (mm^−1^)	9.73
Crystal size (mm)	0.34 × 0.25 × 0.11

Data collection	
Diffractometer	Bruker D8 Venture Diffractometer
Absorption correction	Multi-scan (*SADABS*; Krause *et al.*, 2015 ▸)
*T*_min_, *T*_max_	0.140, 0.259
No. of measured, independent and observed [*I* > 2σ(*I*)] reflections	13169, 1969, 1902
*R*_int_	0.047
(sin θ/λ)_max_ (Å^−1^)	0.609

Refinement	
*R*[*F* ^2^ > 2σ(*F* ^2^)], *wR*(*F* ^2^), *S*	0.024, 0.059, 1.08
No. of reflections	1969
No. of parameters	116
No. of restraints	2
H-atom treatment	H atoms treated by a mixture of independent and constrained refinement
Δρ_max_, Δρ_min_ (e Å^−3^)	0.86, −0.69

Computer programs: *APEX4*, *SAINT* and *XPREP* (Bruker, 2016 ▸), *SHELXT2018/2* (Sheldrick, 2015*a* ▸), *SHELXL2019/2* (Sheldrick, 2015*b* ▸) and *WinGX* publication routines and *ORTEP-3 for Windows* (Farrugia, 2012 ▸).

## supplementary crystallographic information

### Crystal data

**Table:**

[CdBr_2_(C_6_H_14_N_2_O)]	*Z* = 2
*M**_r_* = 402.41	*F*(000) = 380
Triclinic, *P*1	*D*_x_ = 2.568 Mg m^−^^3^
*a* = 7.1291 (2) Å	Mo *K*α radiation, λ = 0.71073 Å
*b* = 7.1662 (2) Å	Cell parameters from 9891 reflections
*c* = 11.0151 (3) Å	θ = 3.0–25.7°
α = 77.704 (1)°	µ = 9.73 mm^−^^1^
β = 80.079 (1)°	*T* = 299 K
γ = 72.371 (1)°	Block, brown
*V* = 520.49 (3) Å^3^	0.34 × 0.25 × 0.11 mm

### Data collection

**Table:**

Bruker D8 Venture Diffractometer	1902 reflections with *I* > 2σ(*I*)
Radiation source: fine focus sealed tube	*R*_int_ = 0.047
φ and ω scans	θ_max_ = 25.7°, θ_min_ = 3.4°
Absorption correction: multi-scan (SADABS; Krause *et al.*, 2015)	*h* = −8→8
*T*_min_ = 0.140, *T*_max_ = 0.259	*k* = −8→8
13169 measured reflections	*l* = −13→13
1969 independent reflections	

### Refinement

**Table:**

Refinement on *F*^2^	Hydrogen site location: mixed
Least-squares matrix: full	H atoms treated by a mixture of independent and constrained refinement
*R*[*F*^2^ > 2σ(*F*^2^)] = 0.024	*w* = 1/[σ^2^(*F*_o_^2^) + (0.0374*P*)^2^ + 0.2615*P*] where *P* = (*F*_o_^2^ + 2*F*_c_^2^)/3
*wR*(*F*^2^) = 0.059	(Δ/σ)_max_ = 0.001
*S* = 1.08	Δρ_max_ = 0.86 e Å^−^^3^
1969 reflections	Δρ_min_ = −0.69 e Å^−^^3^
116 parameters	Extinction correction: *SHELXL2019/2* (Sheldrick, 2015b), Fc^*^=kFc[1+0.001xFc^2^λ^3^/sin(2θ)]^-1/4^
2 restraints	Extinction coefficient: 0.0211 (13)

### Special details

**Table:**

Geometry. All esds (except the esd in the dihedral angle between two l.s. planes) are estimated using the full covariance matrix. The cell esds are taken into account individually in the estimation of esds in distances, angles and torsion angles; correlations between esds in cell parameters are only used when they are defined by crystal symmetry. An approximate (isotropic) treatment of cell esds is used for estimating esds involving l.s. planes.

### Fractional atomic coordinates and isotropic or equivalent isotropic displacement parameters (Å^2^)

**Table:**

	*x*	*y*	*z*	*U*_iso_*/*U*_eq_	
Cd1	0.45454 (3)	0.03786 (3)	0.31347 (2)	0.02454 (11)	
C1	0.2885 (5)	0.5709 (4)	0.4110 (3)	0.0293 (6)	
H1A	0.194661	0.659545	0.462766	0.035*	
H1B	0.346397	0.448474	0.465409	0.035*	
C2	0.1801 (4)	0.5262 (4)	0.3191 (3)	0.0260 (6)	
H2A	0.083408	0.458664	0.364667	0.031*	
H2B	0.109445	0.649989	0.271196	0.031*	
C3	0.4770 (5)	0.4955 (4)	0.1747 (3)	0.0289 (6)	
H3A	0.420658	0.621541	0.123058	0.035*	
H3B	0.573036	0.411330	0.121325	0.035*	
C4	0.5793 (4)	0.5301 (4)	0.2727 (3)	0.0308 (6)	
H4A	0.636222	0.404231	0.324354	0.037*	
H4B	0.686152	0.587572	0.232689	0.037*	
C5	0.2097 (5)	0.3872 (4)	0.1317 (3)	0.0315 (6)	
H5A	0.304928	0.335536	0.064300	0.038*	
H5B	0.132830	0.519547	0.098435	0.038*	
C6	0.0730 (5)	0.2558 (5)	0.1773 (3)	0.0336 (7)	
H6A	−0.019161	0.302997	0.247194	0.040*	
H6B	−0.002850	0.261386	0.110827	0.040*	
N1	0.3180 (3)	0.4002 (3)	0.2323 (2)	0.0230 (5)	
N2	0.1879 (4)	0.0509 (4)	0.2164 (3)	0.0301 (5)	
H2C	0.110 (5)	−0.016 (5)	0.266 (3)	0.036*	
H2D	0.245 (5)	−0.001 (5)	0.150 (2)	0.036*	
Br1	0.72268 (5)	−0.01885 (5)	0.11392 (3)	0.03420 (12)	
Br2	0.76499 (4)	−0.04293 (4)	0.45383 (3)	0.02768 (11)	
O1	0.4421 (3)	0.6610 (3)	0.3494 (2)	0.0302 (5)	

### Atomic displacement parameters (Å^2^)

**Table:**

	*U*^11^	*U*^22^	*U*^33^	*U*^12^	*U*^13^	*U*^23^
Cd1	0.02513 (15)	0.02516 (15)	0.02109 (14)	−0.00408 (9)	−0.00275 (9)	−0.00328 (9)
C1	0.0317 (15)	0.0277 (14)	0.0294 (15)	−0.0084 (12)	−0.0008 (12)	−0.0089 (12)
C2	0.0225 (14)	0.0252 (14)	0.0291 (15)	−0.0048 (11)	−0.0007 (11)	−0.0066 (11)
C3	0.0334 (15)	0.0216 (14)	0.0291 (15)	−0.0100 (12)	0.0058 (12)	−0.0033 (11)
C4	0.0225 (14)	0.0224 (14)	0.0459 (19)	−0.0050 (11)	0.0005 (12)	−0.0074 (12)
C5	0.0458 (18)	0.0260 (14)	0.0236 (15)	−0.0084 (13)	−0.0157 (13)	0.0007 (11)
C6	0.0313 (16)	0.0300 (15)	0.0412 (18)	−0.0040 (13)	−0.0134 (13)	−0.0087 (13)
N1	0.0271 (12)	0.0213 (11)	0.0210 (12)	−0.0079 (9)	−0.0013 (9)	−0.0039 (9)
N2	0.0330 (14)	0.0256 (13)	0.0320 (14)	−0.0100 (10)	−0.0012 (11)	−0.0051 (10)
Br1	0.03434 (19)	0.0396 (2)	0.02838 (19)	−0.01053 (14)	0.00469 (13)	−0.01140 (13)
Br2	0.02187 (17)	0.03721 (19)	0.02266 (17)	−0.00738 (12)	−0.00125 (11)	−0.00454 (12)
O1	0.0294 (11)	0.0236 (10)	0.0397 (12)	−0.0069 (8)	−0.0044 (9)	−0.0098 (8)

### Geometric parameters (Å, º)

**Table:**

Cd1—N2	2.306 (3)	C3—H3A	0.9700
Cd1—N1	2.504 (2)	C3—H3B	0.9700
Cd1—Br1	2.6670 (3)	C4—O1	1.431 (3)
Cd1—Br2^i^	2.7647 (3)	C4—H4A	0.9700
Cd1—Br2	2.7651 (3)	C4—H4B	0.9700
C1—O1	1.436 (4)	C5—N1	1.488 (4)
C1—C2	1.511 (4)	C5—C6	1.508 (4)
C1—H1A	0.9700	C5—H5A	0.9700
C1—H1B	0.9700	C5—H5B	0.9700
C2—N1	1.484 (3)	C6—N2	1.462 (4)
C2—H2A	0.9700	C6—H6A	0.9700
C2—H2B	0.9700	C6—H6B	0.9700
C3—N1	1.481 (3)	N2—H2C	0.886 (18)
C3—C4	1.501 (4)	N2—H2D	0.868 (18)
			
N2—Cd1—N1	76.06 (8)	C3—C4—H4A	109.6
N2—Cd1—Br1	95.75 (7)	O1—C4—H4B	109.6
N1—Cd1—Br1	93.36 (5)	C3—C4—H4B	109.6
N2—Cd1—Br2^i^	93.03 (7)	H4A—C4—H4B	108.1
N1—Cd1—Br2^i^	95.76 (5)	N1—C5—C6	112.6 (2)
Br1—Cd1—Br2^i^	168.635 (14)	N1—C5—H5A	109.1
N2—Cd1—Br2	169.58 (6)	C6—C5—H5A	109.1
N1—Cd1—Br2	113.54 (5)	N1—C5—H5B	109.1
Br1—Cd1—Br2	87.915 (11)	C6—C5—H5B	109.1
Br2^i^—Cd1—Br2	82.231 (10)	H5A—C5—H5B	107.8
O1—C1—C2	112.1 (2)	N2—C6—C5	110.0 (3)
O1—C1—H1A	109.2	N2—C6—H6A	109.7
C2—C1—H1A	109.2	C5—C6—H6A	109.7
O1—C1—H1B	109.2	N2—C6—H6B	109.7
C2—C1—H1B	109.2	C5—C6—H6B	109.7
H1A—C1—H1B	107.9	H6A—C6—H6B	108.2
N1—C2—C1	111.7 (2)	C3—N1—C2	108.2 (2)
N1—C2—H2A	109.3	C3—N1—C5	108.8 (2)
C1—C2—H2A	109.3	C2—N1—C5	109.6 (2)
N1—C2—H2B	109.3	C3—N1—Cd1	111.83 (17)
C1—C2—H2B	109.3	C2—N1—Cd1	118.18 (17)
H2A—C2—H2B	107.9	C5—N1—Cd1	99.75 (16)
N1—C3—C4	111.2 (2)	C6—N2—Cd1	111.49 (18)
N1—C3—H3A	109.4	C6—N2—H2C	109 (2)
C4—C3—H3A	109.4	Cd1—N2—H2C	111 (2)
N1—C3—H3B	109.4	C6—N2—H2D	109 (2)
C4—C3—H3B	109.4	Cd1—N2—H2D	102 (2)
H3A—C3—H3B	108.0	H2C—N2—H2D	114 (3)
O1—C4—C3	110.4 (2)	Cd1^i^—Br2—Cd1	97.768 (10)
O1—C4—H4A	109.6	C4—O1—C1	108.9 (2)
			
O1—C1—C2—N1	55.6 (3)	C1—C2—N1—Cd1	75.7 (3)
N1—C3—C4—O1	−61.5 (3)	C6—C5—N1—C3	168.3 (2)
N1—C5—C6—N2	−64.4 (3)	C6—C5—N1—C2	−73.6 (3)
C4—C3—N1—C2	55.7 (3)	C6—C5—N1—Cd1	51.1 (3)
C4—C3—N1—C5	174.7 (2)	C5—C6—N2—Cd1	37.3 (3)
C4—C3—N1—Cd1	−76.1 (2)	C3—C4—O1—C1	61.1 (3)
C1—C2—N1—C3	−52.6 (3)	C2—C1—O1—C4	−58.5 (3)
C1—C2—N1—C5	−171.1 (2)		

Symmetry code: (i) −*x*+1, −*y*, −*z*+1.

### Hydrogen-bond geometry (Å, º)

**Table:**

*D*—H···*A*	*D*—H	H···*A*	*D*···*A*	*D*—H···*A*
C1—H1*B*···O1^ii^	0.97	2.59	3.370 (4)	138
C3—H3*B*···Br1	0.97	2.96	3.720 (3)	137
C4—H4*B*···Br1^iii^	0.97	2.91	3.678 (3)	137
N2—H2*C*···Br2^iv^	0.89 (2)	2.95 (2)	3.761 (3)	153 (3)
N2—H2*D*···Br1^v^	0.87 (2)	2.86 (2)	3.628 (3)	149 (3)

Symmetry codes: (ii) −*x*+1, −*y*+1, −*z*+1; (iii) *x*, *y*+1, *z*; (iv) *x*−1, *y*, *z*; (v) −*x*+1, −*y*, −*z*.
